# Re-engineering anti-CTLA-4 antibodies for enhancing cancer immunotherapy efficacy and safety

**DOI:** 10.3934/genet.2019.3.64

**Published:** 2019-09-19

**Authors:** Sharvesh Raj Seeruttun

**Affiliations:** Sun Yat-sen University Cancer Center, 651 Dongfeng Road East, Guangzhou 510060, Guangdong, P. R. China

The immune system safeguards the body by controlling the number of circulating/activated immune cells and preventing their over-expression/-activation via co-inhibitory ligands (immune checkpoints) [1]. During normal conditions, these levels are kept at relatively low concentration while in disease conditions, the immune system stimulates their overexpression to combat the disease. All these occur via receptor-ligand interactions that differentiate disease cells to native ones. Cancers are able to progress by presenting receptors mimicking that of normal cells, i.e., tumor cells evade T cell detection by mimicking antigen-presenting cells (APCs) with programmed cell death 1 ligand (PD-L1)—inhibiting ligand, thence escaping immune detection [2]. Immunotherapeutic drugs are molecules administered to the patients to increase the sensitivity of these immune checkpoints, and immunotherapy has thereby revolutionized cancer treatment by reinforcing the host immune cells to target the cancer cells rather than directly targeting the cancer. Promising results have been demonstrated in melanoma, head, and neck, lung, kidney, bladder, colorectal cancer therapy [3]. However, there are still a significant proportion of patients that are non-responsive while those responsive are often afflicted by life-threatening autoimmune-related adverse events (irAEs) [4],[5]. As new immune checkpoint molecules are being investigated in clinical trials, the use of immune checkpoint inhibitors (ICI) would be more common and so irAEs.

irAEs can present at any time and usually develop within the first few weeks to months of treatment initiation. In the neoadjuvant setting of melanoma treatment, combination therapy of ipilimumab to nivolumab resulted in severe irAEs reaching up to 90% [6]. From a more generalized perspective, irAEs occur in nearly 70% and 50% of those treated with anti-CTLA-4 and anti-PD-1/PD-L1 antibodies, respectively, due to the manipulation of the immune system to be more sensitive to these mimicked-receptors that bear high resemblance to innate ones, simultaneously unbalancing the regulatory mechanisms of self-tolerance [7],[8]. As there are still no standardized diagnostic criteria for irAEs, and considering the heterogeneous genetics, epigenetics, and/or microbiota environment existing from patient to patient, the actual prevalence of irAEs in clinical practice may be under-reported [9].

Despite being associated with greater risk of irAEs and that being one of the reasons why till present monotherapy with anti-CTLA-4 have failed in multiple phase III clinical trials, except in melanoma, anti-CTLA-4 antibody was among the first T-cell targeting antibodies to be approved for clinical cancer immunotherapy. Further, treatments offered with anti-CTLA-4 antibodies (Abs) have longer-lasting immunity-effects in cancer patients as compared to anti-PD-1 Abs. However, the underlying mechanisms for these anti-CTLA-4 irAEs remain unclear and thus, in-depth research to improve the efficacy and safety of anti-CTLA-4 Abs are in dire need.

To shed the light on this topic, in a recent publication entitled “Hijacking antibody-induced CTLA-4 lysosomal degradation for safer and more effective cancer immunotherapy”, Zhang et al. [10] investigated the molecular basis for irAEs and cancer immunotherapeutic effects (CITE) of anti-CTLA-4 antibodies. Common practice in immunotherapy is using anti-CTLA-4 Abs to inactivate CTLA-4 on T cells, which prevents it to compete with CD28 to interact with the B7 co-stimulatory ligand on APCs, thereby allowing persistent T cell activation to target cancer cells. However, it is known that the genetic inactivation of CTLA-4 in mice and humans causes severe autoimmune disease [11]. Also, the authors previously reported that blocking the interaction between CTLA-4 and B7 is neither necessary nor sufficient for CITE of anti-CTLA-4 antibodies [12]. As such, the focus should be placed on selective activation of regulatory T cells in the TME rather than in the generalized tissues for improving the mechanism of action of CITE.

Based on these, the authors hypothesized that an antibody-mediated disruption in CTLA-4 recycling might be related to the anti-CTLA-4-induced irAEs. To test this concept, the authors showed that two non-irAEs antibodies failed to downregulate CTLA-4 while two irAEs antibodies (ipilimumab and TremeIgG1) were able to do so as, first, the irAEs-prone Abs were targeted to lysosomes after endocytosis, rather than allowing CTLA-4- lipopolysaccharide-responsive and beige-like anchor (LRBA) interactions for cell surface presentation [2], second, inhibiting the recycling of CTLA-4 molecules in the irAEs-prone Ab-treated cells did not increase the amount of CTLA-4 on the cell surface, and third, the downregulation of CTLA-4 by ipilimumab was inhibited not by a proteasome inhibitor but by a lysosomal degradation inhibitor. Further, the authors also showed that different pH sensitivity anti-CTLA-4 Abs affected differently the CTLA-4 recycling and irAEs in humanized mouse model; simulating that of human irAEs.

Hence, the new approach to increase the treatment efficacy of anti-CTLA-4 treatment, without jeopardizing safety, would be to engineer pH sensitivity into anti-CTLA-4 Abs. For validate this notion, the authors evinced several impactful findings. First, their data demonstrated that pH-sensitive antibodies had superior antibody-dependent cellular cytotoxicity (ADCC) activity than pH-insensitive ones. As ADCC activity is largely dependent on CTLA-4, thereby preservation of CTLA-4 recycling would lead to improve therapeutic of pH-sensitive Abs. Second, although they showed that both pH- sensitive and insensitive Abs could deplete Tregs, pH-sensitive ones were able to do so faster in the TME within 24 h of administration. Third, pH-insensitive anti-CTLA-4 Abs were prone to lysosomal degradation while pH-sensitive ones promoted CTLA-4 recycling, leading to better bioavailability. Fourth, despite both pH sensitivity Abs were able to induce small tumor rejection, pH-sensitive ones were significantly more efficacious and had a 2-fold higher complete large tumor rejection rate.

Through these findings, the author highlighted the mechanisms through which the current approach of using anti-CTLA-4 Abs as cancer treatment can often lead to the clinically observed clinical irAEs and demonstrated the fundamentals for establishing a more efficacious and safer anti-CTLA-4 Abs therapeutic design and identification. As anti-CTLA-4 Abs have longer-lasting immunity in cancer patients than anti-PD-1 Abs, research on improving the efficacy of anti-CTLA-4 Abs should be intensified for better outcomes of cancer immunotherapy.
